# CH5M3D: an HTML5 program for creating 3D molecular structures

**DOI:** 10.1186/1758-2946-5-46

**Published:** 2013-11-18

**Authors:** Clarke W Earley

**Affiliations:** 1 Department of Chemistry, Kent State University at Stark, 6000 Frank Ave. NW, North Canton, OH 44720, USA

**Keywords:** Visualization, Molecular editor, HTML5, 3D, Molecular graphics

## Abstract

**Background:**

While a number of programs and web-based applications are available for the interactive display of 3-dimensional molecular structures, few of these provide the ability to edit these structures. For this reason, we have developed a library written in JavaScript to allow for the simple creation of web-based applications that should run on any browser capable of rendering HTML5 web pages. While our primary interest in developing this application was for educational use, it may also prove useful to researchers who want a light-weight application for viewing and editing small molecular structures.

**Results:**

Molecular compounds are drawn on the HTML5 Canvas element, with the JavaScript code making use of standard techniques to allow display of three-dimensional structures on a two-dimensional canvas. Information about the structure (bond lengths, bond angles, and dihedral angles) can be obtained using a mouse or other pointing device. Both atoms and bonds can be added or deleted, and rotation about bonds is allowed. Routines are provided to read structures either from the web server or from the user’s computer, and creation of galleries of structures can be accomplished with only a few lines of code. Documentation and examples are provided to demonstrate how users can access all of the molecular information for creation of web pages with more advanced features.

**Conclusions:**

A light-weight (≈ 75 kb) JavaScript library has been made available that allows for the simple creation of web pages containing interactive 3-dimensional molecular structures. Although this library is designed to create web pages, a web server is not required. Installation on a web server is straightforward and does not require any server-side modules or special permissions. The ch5m3d.js library has been released under the GNU GPL version 3 open-source license and is available from http://sourceforge.net/projects/ch5m3d/.

## Background

Interactive programs that accurately represent the 3-dimensional structure of molecules are an important tool for modern chemistry. While displaying the structure of biological systems such as proteins is one of the more obvious applications, representation of smaller molecules is also important, particularly in education. To give just a few recent examples, computer graphics has been used by students to visualize the stereochemical nature of molecules [1], crystal structures have been used to illustrate aromatic properties [2], and a web-based tutorial has been developed for understanding pericyclic reactions [3]. A review of approximately 30 apps for visualizing molecular structures on mobile devices was recently published [4].

In 2012, it was noted by the authors of the Avogadro program [5] that “the field of molecular graphics is dominated by viewers with little or no editing capabilities” [6]. While Avogadro certainly does much to rectify this situation, our interest is in providing a web-based tool for students to view and modify small molecules. While students can certainly download and install Avogadro or another stand-alone program to create 3-dimensional structures, most of these students will not be consistently using this type of program for an extended period of time. For this reason, a free or low cost, web-based program was sought that would be usable on a range of platforms. There appears to be very few freely-available web-based programs that provide the ability to interactively view and edit molecular structures.

Graphical programs [7-10] are widely used for the creation of three-dimensional models of compounds to be used as starting points for computational chemistry and for visualization of the results obtained from these calculations. A few of these are freely available web-based programs. WebMO [11] uses the Jmol [12] Java library to build 3-dimensional structures which can then be used as input to quantum mechanical calculations and can display the results of these calculators. Molecular Calculator [13] is a more education-focused application that uses the JSMol [14] library to create web pages that perform many of the same functions as WebMO. Significantly, the JSMol library and Molecular Calculator use HTML5 and do not require Java or any other plugins. However, the emphasis of these programs is on creating input files for quantum mechanical calculations, and both of these programs require installation on a server configured to run PHP. Recognition of the movement toward HTML5 has also been demonstrated by ChemAxon’s recent release of “Marvin for JavaScript” [15], which is a 2-dimensional editor using HTML5 and by Bienfait and Ertl’s release of “JSME” [16], which is a JavaScript 2-dimensional editor based on a conversion of the Java applet JME [17].

Given the fact that Java applets require a separate download, we were most interested in applications using HTML5. In addition to Molecular Calculator, a couple of programs are currently available that use HTML5 to interactively view 3-dimensional structures of molecules. The authors of the JSMol library have created an HTML5 page [14] illustrating some of the capabilities of this library, including the ability to use WebGL. CanvasMol [18] is a demonstration site showing some of the capabilities of HTML5. Fred Ackers [19] has published code combining HTML5 with WebGL to produce a 3D Molecular Viewer. One of the most well-developed HTML5 molecular graphics programs is ChemDoodle [20], which has web components for drawing of two-dimensional structures and viewing of three-dimensional structures. With the exception of Molecular Calculator, these programs do not offer the ability to edit/build 3D molecular structures.

Because of the limited number of options available to suit our needs, we have developed a molecular editor based on HTML5 that requires only an HTML web page, a CSS stylesheet, and one JavaScript library. The primary intended audience for this program is undergraduate students and faculty desiring to use this as an aid for instruction. Documentation is available and several example web pages are included to assist individuals who wish to make use of this library to create web pages that address specific needs.

While not all web browsers currently support HTML5, freely-available web browsers that do support this are available on all major platforms, including most mobile devices. While support for touch screen devices was not a primary design consideration, this program has been tested and works with some limitations on a variety of mobile devices. More complete support is planned to be included in future versions.

## Implementation

### Library development and mouse behavior

The purpose of this program is to provide a browser-based platform for the creation, interactive display, and modification of simple molecules. This package consists of a JavaScript library (ch5m3d.js), a stylesheet (ch5m3d.css), several HTML pages illustrating how this library can be used, a small number of simple “.xyz” files containing molecular coordinates, and a few text files (documentation, license, etc.). The ch5m3d.js library is available under the GNU General Public License, version 3. All other files (web pages, css file, etc.) are not covered under any license and may be freely used or modified. While there are no system-dependent features, this will only run on browsers that support HTML5. This program has been tested and shown to work with recent versions of Microsoft Interenet Explorer (v10), Mozilla Firefox (v19), Google Chrome and Chromium (v22), and Opera (v12). A browser help page is included that performs a simple test for the canvas element and provides links to several freely available web browsers for a range of platforms that are known to work with this application.

All of the code for this library is written in JavaScript, and contains standard routines expected in an interactive molecular graphics program, including the ability to display molecules, label atoms, measure bond distances, measure bond angles, measure dihedral angles, interactively rotate molecules controlled by either buttons or the pointer, highlight select atoms, and rescale (zoom) structures. To the extent possible, all features are available by default with no special coding required within web pages.

Atomic coordinates can be read from files in the XYZ format. This format is described on the OpenBabel website [21]. Every attempt was made to be as flexible as possible in reading these files, and spacing/column alignment is not crucial. Note that this format does not include any bond connectivity information. Upon successful reading of atomic coordinates, interatomic distances for all pairs of atoms are calculated, and if this distance is less than 1.2 times the sum of the covalent radii for these atoms, a bond is created. No attempt is made to determine the bond order in the current version of this library. If this capability is added in future versions, it will be necessary to add the ability to read and write files in a format that includes connectivity information. Preliminary support for reading MDL’s MOL format (which includes bonding information) is implemented in this library.

The HTML5 Canvas is used to display “ball-and-stick” structures of molecules with the size of atoms based on their covalent radii. It is assumed that atomic spheres do not overlap. Because the canvas element is two-dimensional, atoms are sorted by depth and drawn from back-to-front. To draw a molecule, the backmost atom is drawn using the “arc” element and filled using radial gradients to give the illusion of depth for each sphere. Colors for all elements are the same as those used in the Jmol [12] program. Bonds are drawn from this sphere to all connected atoms in front of this atom as lines originating from the point of intersection of the surface of the sphere and the bond for the back atom and terminating at the atomic center for the front atom. This process is then repeated until all atoms and bonds in the compound have been drawn.

This library was designed to be used with small molecules, and very few attempts were made to optimize this program for speed. No limits are placed on the array dimensions in this library, so the number of atoms that can be displayed is technically only limited by available memory. While structures containing a few thousand atoms can easily be viewed, it takes a noticeable amount of time to display large structures, and interacting with compounds of this size (rotations, etc.) is too slow to be practical. Because display and editing of large molecules is not necessary for the primary intended audience, it was not considered worthwhile to include WebGL in the initial release of this library. Implementation of WebGL would enable hardware-acceleration and allow larger molecules to be visualized, and it is possible that this will be added in future versions of this library. One simple technique has been implemented to optimize the display of larger molecules. Implementation of radial gradients is computationally expensive. For this reason, atoms are drawn without gradients in molecules containing over 250 atoms.

User interaction with molecules is accomplished using the mouse or related pointing device. Event listeners are set up for common mouse and touch events (mouse down, mouse up, mouse move, and mouse wheel). The action resulting from triggering of these events depends on the currently active 'mode’. It was decided early in development to define separate “View” and “Draw” modes. In View mode, the molecule cannot be changed, so mouse behavior is limited to rotation of the entire molecule or selecting atoms to provide information. Rotations around the x- and y-axes are performed by dragging the mouse pointer, starting on a blank portion the active canvas. Simultaneously pressing the [Shift] key allows rotation around the z-axis, while pressing the [Ctrl] key allows translations in the xy-plane. Rotation angles are based on the amount of horizontal and vertical movement of the mouse, with new molecular coordinates calculated based on standard matrix transformations. Selecting an atom provides information about this atom (elemental symbol and position in molecular coordinate list by default). If additional atoms are selected, bond lengths, angle, and dihedral angles can be viewed. Selecting an atom while pressing the [Shift] key toggles highlighting of the selected atom.

In Draw mode, the molecule can be changed, and the mouse is used to add, delete, or change atoms and to add or delete bonds. Switching between View and Draw modes is typically controlled by the user selecting a button which calls either the viewmode() or drawmode() function. The default behavior in Draw mode is to add atoms or bonds. By default, carbon atoms will be added, but this can be changed by a call to the pickElem function passing the symbol of the desired element as the parameter. For example, to change to adding nitrogen atoms, the function call would be: 

To aid in selecting elements, a function is called during initialization that looks for a division within the web page with an id of 'ptable’. If this division is found, this function will write the HTML code necessary to create a periodic table with each elemental symbol defined as a button that makes the appropriate call to the pickElem() function when selected. By default, only main group elements are shown initially, but a full periodic table is also available to allow any element to be selected, which allows building of both organic and inorganic structures.

In general, selecting an individual atom changes that atom into whichever element is currently active. If the atom selected has two or more bonds, only the element type is changed. If the atom has only one bond, then the selected atom is deleted and the new atom added at a distance set as the sum of the covalent radii of the bonded atoms. For p-block elements only, the program will also attempt to add a suitable number of hydrogen atoms at appropriate positions. Bond lengths for these hydrogen atoms are determined based on covalent radii, and bond angles are determined by the user selectable hybridization, which by default is set as sp^3^. The number of hydrogen atoms to add (up to a maximum of three) is determined assuming that atoms obey the octet rule and is calculated from the equation: 

If the active element is hydrogen, a different procedure is used. Selecting any atom will attempt to add a single hydrogen atom to this atom at a bond distance determined by the covalent radii and in a position intended to minimize interactions with other bonded atoms. Because no other atoms are moved, the result of this action is often not ideal. A very crude geometry optimization routine has been implemented, but this code is not optimized and is being actively developed to improve its speed and performance. This optimization is not a true molecular mechanics optimization, but simply optimizes bond lengths based on covalent radii, bond angles based on simple VSEPR theory, and minimizes torsional stress by changing dihedral angles.

In Draw mode, rotation of the entire molecule can be performed by “dragging” the pointer starting from a blank portion of the drawing canvas. Rotation about the z-axis is accomplished by holding the [Shift] key down during the rotation. Rotation of fragments about bonds is also possible as long as the selected bond is not part of a ring. Bonds can be added by holding the mouse pointer down on one atom and dragging the pointer to a second atom. If mistakes are made, an “Undo” function is available. This has been implemented as a simple stack, so a limited number of both “Undo” and “Redo” operations (currently 10) are possible.

To change the mouse behavior to delete atoms or bonds, a call is made to the pickElem function with a parameter of 'Delete’ or 'DelBond’. When deleting atoms, attached hydrogen atoms are also removed. To return back to the add atoms/bonds mode, pickElem is called with the desired elemental symbol.

Multiple molecules can be displayed on the same web page, and there is no hard-coded limit to the number of windows that can be placed on each page. The only requirement is that each window has a unique 'id’. The activeWin function associates a set of molecular coordinates with a canvas, and is used by all functions controlling the display of molecules, including all mouse/pointer functions. “Mouse” events are defined for each canvas, so “clicking” anywhere on any canvas can be used to set the active canvas for all user input. As a result, a single set of user interface controls (such as buttons) may be used regardless of the number of canvas elements placed on a page.

### Installation

One of the design goals of this project was to make installation of this program as simple as possible. The program is distributed as a compressed zip archive. After unpacking this in any convenient location, a web browser can be used to view the default web page (index.html) which provides access to the documentation and several variations providing examples of different ways this library can be used. It is not required install this on a web server. Minimally, the only required files are the ch5m3d.js JavaScript library, the ch5m3d.css stylesheet, and an HTML file to load these files and create the HTML5 Canvas element along with the interface elements required to load, view, and edit files. The default page includes a toolbar linking to documentation files, license information, and the project website.

Installation on a web server requires copying the unpacked archive to a location that can be read by the server. No modifications of any of these files should be required, and the only permission required is read access. In some installations, it might be convenient to create a link to this directory.

## Results and discussion

### Included web pages

Several fully functional, sample web pages are included with this package that illustrate different ways that HTML pages can access this library. In the root directory of this package is a default web page named index.html, which demonstrates most of the main features of this library. By default, an image of the methane (CH_4_) molecule is displayed. A “browse” button is provided to allow the user to load files in the.xyz format, and a few sample files are included in the molecules subdirectory.

**Figure 1:**
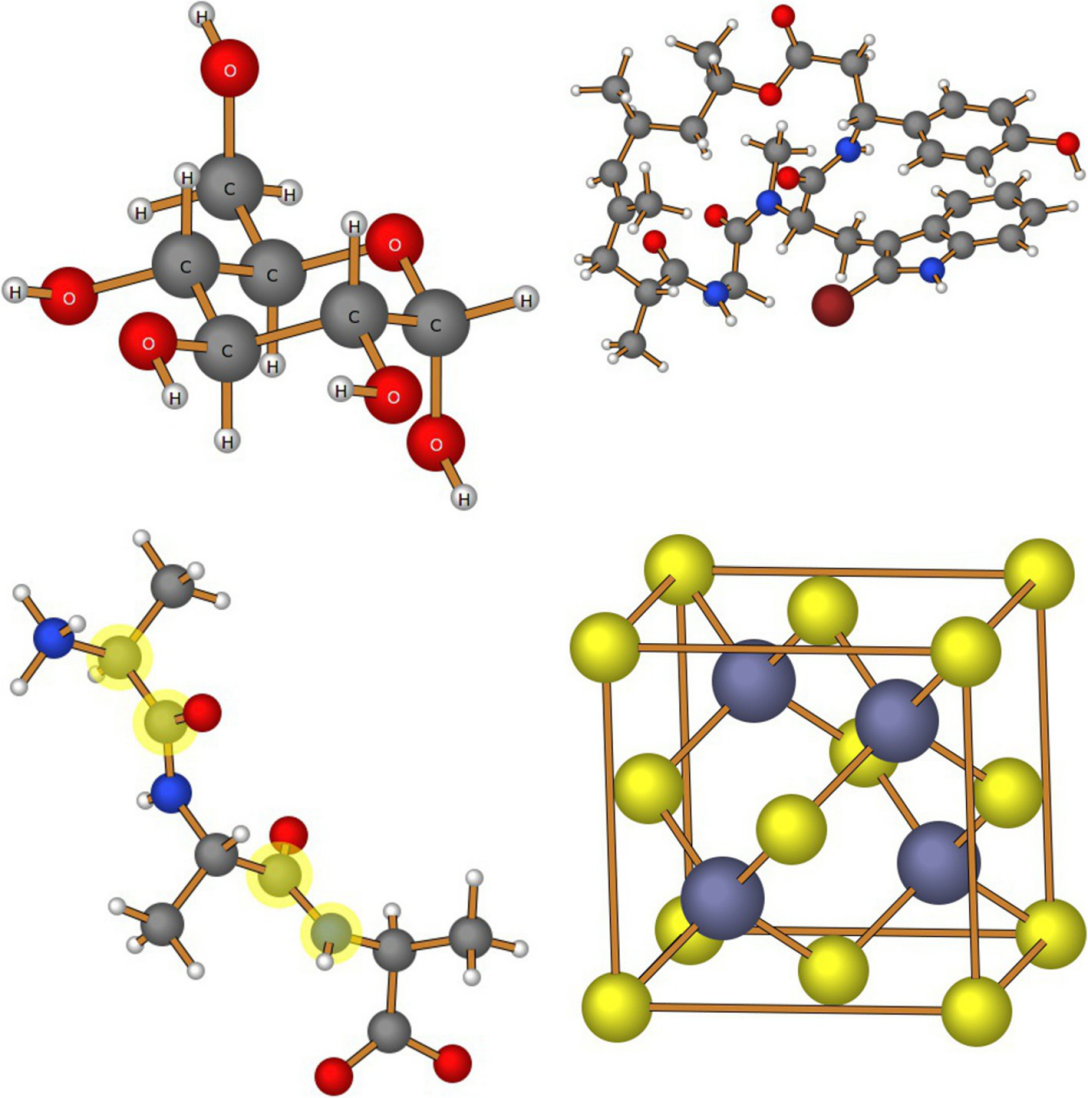
**Sample molecules.** Molecular images generated using the CH5M3D library.

Figure 1 provides an illustration of images created by this program. The display of atomic labels, as shown in the structure of D-glucopyranose (upper left), is accomplished using the “Labels” button. Individual atoms, such as the carbon and nitrogen atoms of peptide bonds, can be highlighted (in light yellow) by holding down the [Shift] key when selecting an atom. In the image of zinc blende (lower right), “bonds” were created between the eight corner atoms to emphasize the unit cell in this structure.

A number of additional web pages are included in a subdirectory named “variations” that illustrate different ways that this library can be used. These include: 

• **Pre-load**: This simple page loads and displays the structure of a molecule from a file stored on the server. The name of this file is part of the web page HTML and cannot be changed by the user. While the molecule can be rotated and information displayed, the user cannot change this structure.

• **Chooser**: This page allows the user to select the file to be viewed from a list of files stored on the server using either buttons or from a drop-down select list. While the molecule can be rotated and information displayed, the user cannot alter any of these structures.

• **Gallery**: This page loads a list of files stored on the server and displays each of these in a separate division along with descriptions. Each of the molecules can be rotated independently and information displayed. However, the user cannot change any of these structures.

• **Viewer (only)**: This page allows loading and viewing of molecules from files stored on the user’s computer, but does not allow for any editing of these structures.

• **View 2 Windows**: This page illustrates that more than one molecule can be loaded on a page. This page also does not allow for any editing of either structure. To switch between active windows, use the mouse to click on any portion of a drawing canvas.

• **Two Windows**: This page illustrates that more than one molecule can be loaded on a page, and that these windows do not have to be the same size. In this view, both View Mode and Draw Mode are enabled, so either (or both) of the structures being displayed can be altered.

• **JavaScript**: This page illustrates how a user might create a simple function that interacts with functions contained within the CH5M3D library to gather information about the active molecule and interact with this structure. In this example, mirror images of a chiral molecule are generated with the user selecting which mirror plane to use.

### Creating web pages

This section outlines the basis steps required to create a functioning web page using the CH5M3D library. All of the JavaScript required to use this program is contained in the external library ch5m3d.js. The layout and appearance of this editor is controlled using the ch5m3d.css stylesheet. Typically, both of these files will be stored in the same directory as the HTML web pages and included in the HEAD section of the web page using the following statements. 

**Figure 2:**
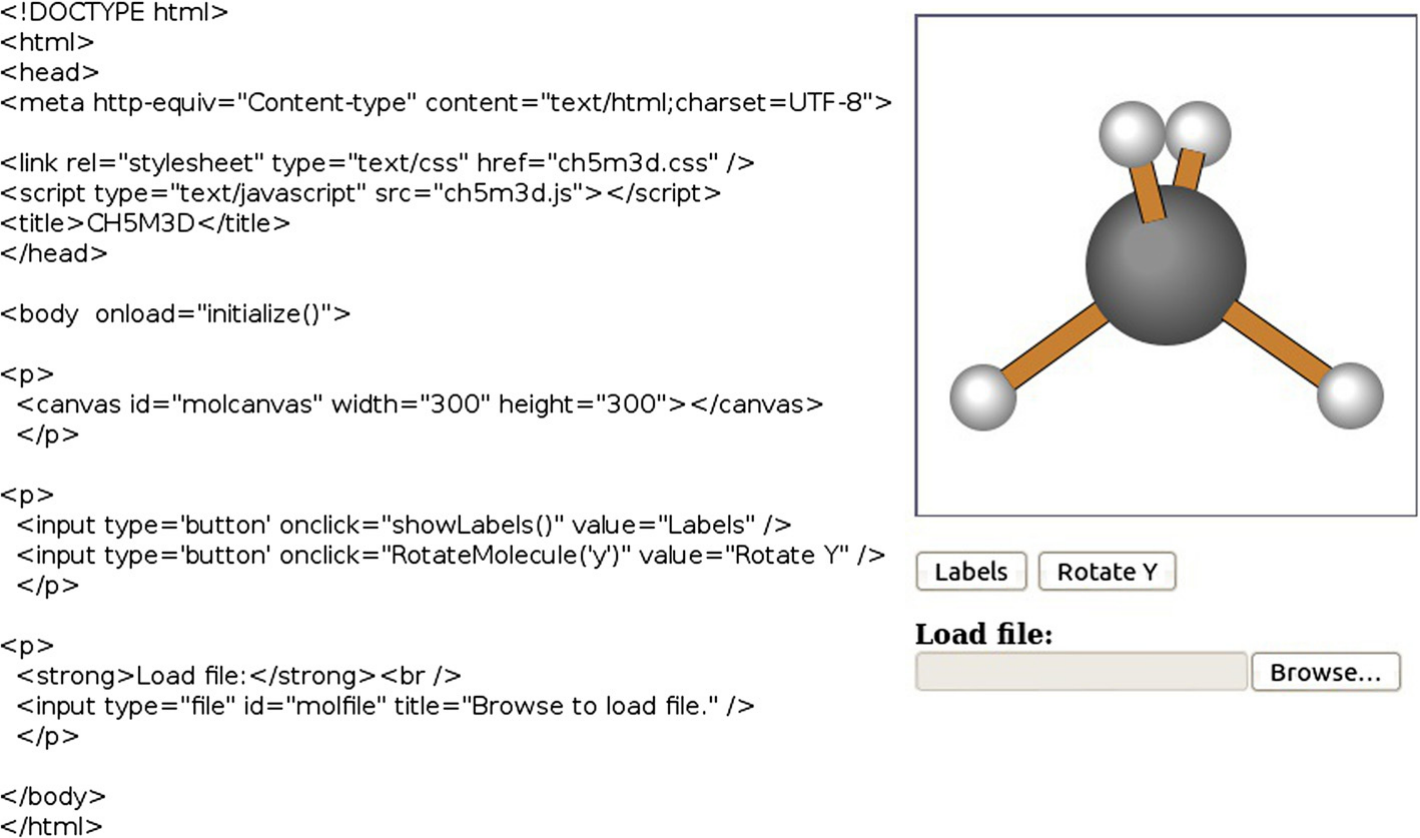
**Simple viewer.** The complete HTML code listing required to provide a simple interface without structure editing capabilities.

 Many of the CH5M3D library routines need to be run after the page has been read by the browser. This can be accomplished by adding the following line to the < body > statement: 

As illustrated in Figure 2, the HTML code required to create simple web pages is minimal. The most important HTML5 element required to use this program is the Canvas object, which provides the drawing window for showing the molecule. This element must be given a unique identifier using the “id” tag. The dimensions of this canvas are set by the HTML declaration, and can be adjusted as desired. While a square canvas is usually preferred, the width and height are not required to be identical. 

This is all the information required to create an interactive display, which by default will display the structure of the methane molecule. The first feature necessary to make this a useful application is the ability to load and view different structures. In some situations, it might be preferred to automatically load specific 3-dimensional molecular coordinates from files stored on the web server, while in other cases users could be given the option to load files stored on their computer. Currently, only.xyz files (such as those created by Open Babel [21]) containing cartesian coordinates are supported. A small selection of.xyz formatted files is available from the project home page, and the format of these files is described both in the documentation and on the Open Babel website.

Reading files from the user’s computer is accomplished using the HTML5 FileReader function. The ch5m3d.js initialize function creates a “listener” for an object with an id of “molfile”. When the user selects a file, the change in this object is detected and the appropriate routines are called to read and display the molecule. To implement this type of file reader on a web page, a statement similar to the following can be used, which will create a familiar “Browse” button allowing users to select any file stored on their computer. No filtering is performed, so users can select any type of file, including files that do not contain molecular coordinates and files in unsupported formats. Files with an incorrect extension are ignored with a warning message to the user. 

It is also possible to read molecular coordinate data from files stored on the web server. This can be accomplished by using the CH5M3D function readServerFile(*filename*), which gets the contents of the file using the XMLHttpRequest() JavaScript function. The path to the filename sent to the readServerFile file is relative to the location of the web page. The filename sent to this function will generally be coded into the web page. This can be done in a manner that allows user interaction by using buttons or a select list. Alternatively, a simple JavaScript function can be created that will allow this file to be loaded automatically when the web page is loaded by the browser. Examples of these methods are included with this program. To aid in creation of web pages displaying multiple windows, a “showgallery” function is defined. The key parameters for this function are an array of filenames and a matched array containing a description of each molecule.

### User-defined functions

By default, this library provides the necessary tools to allow for viewing and modification of molecular structures. It is also possible to use this library to create customized pages to address specific needs. The documentation included with this program (“Library API Info”) includes a brief description of functions accessible to users from their own JavaScript routines. The last function call of the CH5M3D 'initialize()’ function is to 'userDefined()’, which provides a convenient hook to allow users to add additional JavaScript code that will run whenever the web page is loaded. One obvious use for this function is to automatically read molecular coordinates and display one or more structures when a web page is loaded. For example, to automatically load the file adamantane.xyz in the molecules subdirectory (relative to the directory containing the web page), the following code could be used. 

The file api.html (located in the variations subdirectory) is a more complicated example that illustrates how molecular information can be obtained within JavaScript. This example initially loads coordinates for the S-2-butanol molecule and displays this structure. The number of atoms in this structure is determined, then the molecular weight and coordinates for all non-hydrogen atoms are displayed in the information box. Buttons are provided to allow the user to select a mirror plane. Pressing one of these buttons calls a second function that calculates and stores coordinates for the mirror image of the molecule and displays this new structure on a second canvas.

## Conclusions

We have described development of a library that allows for the easy creation of web pages providing the ability to display and edit both organic and inorganic molecular structures. Installation is simple and does not require any special permissions on the web server. Users benefit from not having to install any additional software, and the web pages are viewable on a wide range of browsers across different operating systems. While this library does not include as many features as more full-featured applications such as Jmol, this library is relatively small (≈ 75 kb) and can easily be extended to provide a range of capabilities. While the most recent version of this program is available on the project homepage, a demonstration of this program is available at the end of the full-text HTML version of this article [Additional file 1].

## Availability and requirements

**Project Name:** CH5M3D**Project home page:** http://sourceforge.net/projects/ch5m3d/**Operating system(s):** Platform independent**Programming language:** JavaScript**Other requirements:** Web browser supporting HTML5, Web server (optional)**License:** GNU GPL v3**Any restrictions to use by non-academics:** None

## Competing interests

The author declares that he has no competing interests.

## Supplementary Material

Additional file 1 This archive contains all of the files required to create a fully-functional website using the CH5M3D library. Click here for file
