# Supplementary material for: CH5M3D: an HTML5 program for creating 3D molecular structures
Source: J Cheminform. 2013 Nov 18;5:46. doi: 10.1186/1758-2946-5-46 (PMC4177146; doi:10.1186/1758-2946-5-46)
Supplement: Additional file 1 — This archive contains all of the files required to create a fully-functional website using the CH5M3D library. [file 1758-2946-5-46-S1.zip › ch5m3d/qchem/qchem.php]

 require\_once "auth.php" ?

CH5M3D


CH5M3D

- CH5M3D Home
- Documentation
  - Introduction
  - Installation
  - Web Browsers
  - User Interface
  - Keyboard/Mouse
  - Drawing
  - File Format
  - PDF Manual
- Variations
  - Description
  - Pre-Load
  - Chooser
  - Gallery
  - Viewer (only)
  - View 2 Windows
  - Two Windows
  - Javascript
  - Quantum Interface
- Information
  - About
  - Project Homepage
  - Library API Info
  - GNU License
- Welcome  echo $\_SERVER['PHP\_AUTH\_USER'] ?

**Local files:**

**Server files:**

Permanently delete files:

**Server Files**

**View Mode**

Formula =

**Current Element:**

|  |  |  |  |  |  |  |  |  |  |  |  |  |  |  |  |  |  |
| --- | --- | --- | --- | --- | --- | --- | --- | --- | --- | --- | --- | --- | --- | --- | --- | --- | --- |
| H |  | | | | | | | | | | | | Organic | | Metals | | He |
| Li | Be |  | | | | | | | | | | B | C | N | O | F | Ne |
| Na | Mg |  | | | | | | | | | | Al | Si | P | S | Cl | Ar |
| K | Ca | Sc | Ti | V | Cr | Mn | Fe | Co | Ni | Cu | Zn | Ga | Ge | As | Se | Br | Kr |
| Rb | Sr | Y | Zr | Nb | Mo | Tc | Ru | Rh | Pd | Ag | Cd | In | Sn | Sb | Te | I | Xe |
| Cs | Ba | La | Hf | Ta | W | Re | Os | Ir | Pt | Au | Hg | Tl | Pb | Bi | Po | At | Rn |
| Fr | Ra | Ac | Rf | Db | Sg | Bh | Hs | Mt | Ds | Rg | Cn | Uut | Fl | Uup | Lv | Uus | Uuo |
|  | | Ce | Pr | Nd | Pm | Sm | Eu | Gd | Tb | Dy | Ho | Er | Tm | Yb | Lu |  | |
|  | | Th | Pa | U | Np | Pu | Am | Cm | Bk | Cf | Es | Fm | Md | No | Lr |  | |

**Hybridization:**

### Parameters for quantum mechanical calculation

Name for input file:

  
Comment:

  
Charge:

  
Type of Calculation:

Single-Point Energy      
Geometry Optimization      
Optimization and Frequency
  
Basis Set:

STO-3G      
3-21G      
6-31G\*      
6-311G\*\*
  
DFT Functional:

None      
B3LYP      
B97-D      
PBE0      
M11      
wB97X-D      
  
PCM Solvent:

None      
Water      
Methanol      
Ethanol      
Acetone      
THF

You may edit the data in the **Information** box below.
When finished, submit the job by pressing the [Start Calculation] button.

**Information**

The chem3d.js library copyright © 2013 by Clarke Earley  
and is distributed under the terms of the
GNU General Public License.
