# Supplementary material for: CH5M3D: an HTML5 program for creating 3D molecular structures
Source: J Cheminform. 2013 Nov 18;5:46. doi: 10.1186/1758-2946-5-46 (PMC4177146; doi:10.1186/1758-2946-5-46)
Supplement: Additional file 1 — This archive contains all of the files required to create a fully-functional website using the CH5M3D library. [file 1758-2946-5-46-S1.zip › ch5m3d/doc/keyboard.html]

CH5M3D


CH5M3D

- CH5M3D Home
- Documentation
  - Introduction
  - Installation
  - Web Browsers
  - User Interface
  - Keyboard/Mouse
  - Drawing
  - File Format
  - PDF Manual
- Variations
  - Description
  - Pre-Load
  - Chooser
  - Gallery
  - Viewer (only)
  - View 2 Windows
  - Two Windows
  - Javascript
  - Quantum Interface
- Information
  - About
  - Project Homepage
  - Library API Info
  - GNU License

# User Interface: Keyboard and Mouse

While most common operations can be performed using only a mouse, a few operations require a
combination of both keyboard and mouse. Note that these operations only work in **View Mode**.
Note that in these descriptions, the screen is assumed to show the X-axis (horizontal) and the Y-axid (vertical),
with the Z-axis coming "out" of this plane. The possible combinations and their results are outlined below.

- Selecting a blank portion of the screen:
  - **Mouse only** - Dragging the mouse results in rotating the molecule around the
    X- and Y-axes.
  - **<Shift> + Mouse** - Rotation will occur around the Z-axis.
  - **<Ctrl> + Mouse** - The molecule will be translated along the X- and Y-axes.- Selecting a single atom:
    - **Mouse only** - The label for this atom is shown. (If the charges button is pressed,
      the calculated charge on this atom is also shown).
    - **<Shift> + Mouse** - The selected atom is highlighted. If this atom is already highlighted,
      this operation will cause the highlight to be removed.

A
button is provided that will allow automatic re-scaling and re-centering of the molecule. This button will also
remove all highlights from the displayed structure.

The chem3d.js library copyright © 2013 by Clarke Earley  
and is distributed under the terms of the
GNU General Public License.
