# Supplementary material for: CH5M3D: an HTML5 program for creating 3D molecular structures
Source: J Cheminform. 2013 Nov 18;5:46. doi: 10.1186/1758-2946-5-46 (PMC4177146; doi:10.1186/1758-2946-5-46)
Supplement: Additional file 1 — This archive contains all of the files required to create a fully-functional website using the CH5M3D library. [file 1758-2946-5-46-S1.zip › ch5m3d/doc/fileformat.html]

CH5M3D


CH5M3D

- CH5M3D Home
- Documentation
  - Introduction
  - Installation
  - Web Browsers
  - User Interface
  - Keyboard/Mouse
  - Drawing
  - File Format
  - PDF Manual
- Variations
  - Description
  - Pre-Load
  - Chooser
  - Gallery
  - Viewer (only)
  - View 2 Windows
  - Two Windows
  - Javascript
  - Quantum Interface
- Information
  - About
  - Project Homepage
  - Library API Info
  - GNU License

# File Format for Molecules

Currently, the only format supported by this program for reading molecular coordinates is the .xyz format.
These files can be created using the Open Babel program. Sample files
in this format are available in the molecules directory.

### Example

On the default page is a
button that displays coordinates for the current molecule in the information box at the bottom of the
screen. An example of the output produced for the methane molecule is:

```
5
CH4   (16.04 g/mol) in xyz format: From CH5M3D
C         0.0000       0.0000       0.0000
H         0.8740       0.6180       0.0000
H        -0.8740       0.6180       0.0000
H         0.0000      -0.6180       0.8740
H         0.0000      -0.6180      -0.8740
```

The first line contains the number of atoms in the molecule.

The second line is a comment. This line must be present, but can be blank or contain any text.

The remaining lines contain the elemental symbol (1-3 characters) and x, y, z cartesian coordinates. All of these
values are separated by one or more spaces.

This program attempts to be flexible in reading these files, and does not require data to be present in specific
columns. The one restriction that is enforced is that files must be named with the extension ".xyz".

The chem3d.js library copyright © 2013 by Clarke Earley  
and is distributed under the terms of the
GNU General Public License.
