# Supplementary material for: CH5M3D: an HTML5 program for creating 3D molecular structures
Source: J Cheminform. 2013 Nov 18;5:46. doi: 10.1186/1758-2946-5-46 (PMC4177146; doi:10.1186/1758-2946-5-46)
Supplement: Additional file 1 — This archive contains all of the files required to create a fully-functional website using the CH5M3D library. [file 1758-2946-5-46-S1.zip › ch5m3d/doc/documentation.book.html]

CH5M3D


# About CH5M3D

**CH5M3D Version 1.2.5**

Project Homepage

### Description

This program uses a combination of HTML5 and javascript to interactively draw 3-dimensional
structures of small molecules.

### License

This program is free software: you can redistribute it and/or modify it under the terms of the
GNU General Public License as published by the Free Software Foundation, either version 3 of the
License, or (at your option) any later version.

This program is distributed in the hope that it will be useful, but WITHOUT ANY WARRANTY; without even
the implied warranty of MERCHANTABILITY or FITNESS FOR A PARTICULAR PURPOSE. See the
GNU General Public License for more details.

To view a copy of the GNU General Public License, see
http://www.gnu.org/licenses/gpl.html.

### Author

Dr. Clarke Earley  
Associate Professor of Chemistry  
Kent State University at Stark  
cearley@kent.edu

### Acknowledgements

The support of Kent State University at Stark is gratefully acknowledged.

# CH5M3D Overview

This web interface has been developed to allow users to create and visualize 3-dimensional drawings of simple
molecules without requiring the download of any additional software. In addition to creating structures, users
can also load existing XYZ formatted files (such as those generated by
Open Babel) containing 3-dimensional coordinates. A small
selection of .xyz formatted files is available from the
project home page.

Once molecules are created or read from a file, users can examine geometry information (bond lengths, angles,
and dihedral angles) and modify these structures. Routines to perform a crude geometry optimization and a simple
calculation of atomic charges are also available.

This web interface relies on HTML5 (in particular, the HTML5 Canvas), and as such requires a
modern web browser to run. The advantage of this approach is the no additional
plugins or Java is required to use this program. Support for mobile devices is currently only partially implemented,
but is planned to be included in future versions.

### Screenshots

Initial view of CH5M3D Interface

Image with labels added

Image with highlighting

Draw Mode interface

Example of charge calculation

# Installation

This "program" can be downloaded as a single zip file containing the following files.

- **index.html** - The main HTML file loaded by a web browser.
- **ch5m3d.js** - The javascript code required to generate the web interface.
- **ch5m3d.css** - CSS code controlling the appearance of the web pages.
- **documentation.pdf** - A PDF version of the documentation.
- **doc/** - A directory containing html documentation files and the GNU license.
- **molecules/** - A directory containing a small number of .xyz formatted
  files of molecular coordinates.
- **variations/** - A directory containing a few html files that illustrate different ways
  that web pages can use the javascript code to provide different views.
- **qchem/** - A directory containing a php files and support files that provide a simple
  front-end interface to the quantum mechanical program GAMESS. (Note that it is very unlikely that this
  will work without modification).

### Testing

A live version of this program is available at the
Project Web page on SourceForge. Note that a
suitable web browser that supports HTML5 is required to run this program.

### Installation on a Web Server

No special steps are required to install this program on a web server other than placing the "unzipped"
directory tree in a location where it can be accessed by the web server. All file locations are coded
as "relative" directories, so the actual path/directory location for these files does not matter.

If it is desired to change the relative location of any of these files, note that **index.html**
assumes that **ch5m3d.js** and **ch5m3d.css** are both located in the same directory
as **index.html**. All of the documentation files assume that **ch5m3d.css**
is located "up" one directory (../ch5m3d.css).

# Supported Web Browsers

The interactive drawing window used on this website requires HTML5, which is NOT supported by version 8
or lower of Microsoft Internet Explorer. To use this site, you will need to use a browser that
supports the Canvas element and a few other features of HTML5. Listed below are several freely available
web browsers that should work with this interface.

- Mozilla Firefox - Available for Microsoft Windows,
  Apple, Linux, and Android.
- Google Chrome - Available for Microsoft Windows,
  Apple, Linux, Android, and iOS.
- Opera - While not as popular, is available for Microsoft
  Windows, Apple, Linux, Android, and iOS.
- Apple Safari - Should already be installed on current Mac
  computers.

Currently, touch screen devices are NOT fully supported by this interface. While the web page will load, several
important tasks do not function properly on tablets or other touch screen devices.

# User Interface: View Mode

The image below shows the initial view that should be presented when this page is first loaded.
The most important sections are labeled in red. Upon initially loading this page, you should be
in **View Mode**, indicated by both the highlighted
**[View Mode]**
tab and the text "View Mode" displayed below this button. You can switch between
**View Mode** and **Draw Mode** at any time by selecting the appropriate tab.

Initially, the methane molecule (CH4) should be displayed in the
**Drawing Window**. While in View mode, you can use your mouse/pointer to perform the
following tasks.

- **Rotate Molecule** - Move the pointer to any blank portion of the Drawing Window
  and hold the mouse button down. Dragging the pointer should cause the molecule to rotate. To stop
  rotation, simply release the mouse button. Simultaneously pressing **<Shift>**
  while dragging the mouse pointer will cause the molecule to rotate around the z-axis. It is
  also possible to 'translate' a molecule by pressing the **<Ctrl>** key
  while dragging the mouse.
- **Zoom** - If a mouse scroll wheel is available, this can be used to either zoom-in
  or zoom-out the current view.
- **Identify Atom** - Move the pointer over any atom in a molecule and click on this
  atom to select. The Elemental symbol followed by a number should be displayed. The number is the
  position of the atom in the set of coordinates for this molecule, starting at 1. If the
   **[Charges]**
  button is active, then the calculated charge on this atom will also be displayed.
- **Highlight Atom** - Move the pointer over any atom in a molecule, hold down the
  **<Shift>** key and click on this atom. A semitransparent yellow circle should
  appear over this atom. <Shift>-click on this atom a second time to turn off the highlight.
  Any number of atoms may be highlighted. The
   **[Reset View]**
  button may also be used to remove all highlights.
- **Measure Bond Lengths** - Move the pointer to the first atom of the bond and select.
  The label for this atom should appear in the upper-right corner of the display window. Then, move
  the pointer to the second atom and select this atom. The bond length (in Angstroms) will be shown
  at the top right of the drawing window.
- **Measure Bond Angles** - By selecting three atoms, the bond angle (in degrees)
  around the central atom (the second atom selected) will be shown.
- **Measure Dihedral Angles** - By selecting four atoms, the dihedral angle
  (in degrees) will be shown. For example, if all four atoms lie in a plane, the dihedral angle will
  be either 0° or 180°.

For all of the operations, clicking on a blank portion of the screen will clear the list of selected
atoms and allow you to start measuring a different set of lengths/angles. Clicking on the same atom
twice will cause the atom list to be reset and this selected atom will be placed as the first atom on
the list.

To the right of the **Drawing Window** are several buttons. These provide the following
options:

- **[Show coordinates]**
  This button will print the coordinates in .xyz format in the **Information Window**.
  For security reasons, it is not possible for javascript to write files to a user's computer.
  To save the coordinates of molecules created using this program, it is required that users open a
  text editor, copy the coordinates from the **Information Window** into their editor
  and save this as an .xyz file. When saving this file, but sure that it is saved as an unformatted
  text file.
- **[Labels]**
  This button toggles the display of labels (elemental symbols) for each atom.
- **[Charges]**
  Selecting this button causes a crude computation of atomic charge to be performed. Charges are shown
  as semi-transparent spheres around each atom. Negative charges are shown in red, and positive charges
  are shown as blue spheres. The intensity of these colors is used to indicate the magnitude of the charges.
  This charge calculation is based on a combination of formal charges and electronegativity differences.
  In addition, the dipole moment of the molecule based on these approximate point charges is displayed in
  the Information window near the bottom of the screen. Note that these calculated charges should *not*
  be considered to be accurate values but instead treated as a crude, first approximation.
- **[Reset View]**
  This button centers and rescales the molecule to fit the display, and removes all highlights.
- **[Browse...]**
  By selecting this button, users can load .xyz formatted files. Files of this format can be generated
  using the Open Babel program. A few sample .xyz files
  are available in the **molecules** folder on the
  CH5M3D Web site.

To **save an image** of a molecule, first rotate the molecule to get it into the desired
orientation. Then, perform a right-click using the mouse pointer and select "Save Image As...".

# User Interface: Keyboard and Mouse

While most common operations can be performed using only a mouse, a few operations require a
combination of both keyboard and mouse. Note that these operations only work in **View Mode**.
Note that in these descriptions, the screen is assumed to show the X-axis (horizontal) and the Y-axid (vertical),
with the Z-axis coming "out" of this plane. The possible combinations and their results are outlined below.

- Selecting a blank portion of the screen:
  - **Mouse only** - Dragging the mouse results in rotating the molecule around the
    X- and Y-axes.
  - **<Shift> + Mouse** - Rotation will occur around the Z-axis.
  - **<Ctrl> + Mouse** - The molecule will be translated along the X- and Y-axes.- Selecting a single atom:
    - **Mouse only** - The label for this atom is shown. (If the charges button is pressed,
      the calculated charge on this atom is also shown).
    - **<Shift> + Mouse** - The selected atom is highlighted. If this atom is already highlighted,
      this operation will cause the highlight to be removed.

A
 **[Reset View]**
button is provided that will allow automatic re-scaling and re-centering of the molecule. This button will also
remove all highlights from the displayed structure.

# Drawing Molecules

In Draw Mode, you have the option of adding or deleting atoms and/or bonds to any structure shown in the
display window. The image below shows the buttons initially displayed when first entering Draw Mode.

### Rotating the Entire Molecule

In draw mode, you can rotate the molecule in the same manner as performed in View mode by selecting a
blank portion of the screen and "dragging" the pointer.

### Adding Atoms

Initially, a subset of the periodic table is shown, with Carbon highlighted. To add a methyl group to the
methane molecule shown, click on any of the hydrogen atoms. By default, the atom added is assumed to be
sp3 hybridized. To add a CH2 group, select
 **[sp2]**
before clicking on an H.

In general, clicking on any atom will convert the atom into the selected element type shown on the
periodic table. If the selected atom has only one bond, the new atom is added with a reasonable value
for the bond length and the appropriate number of hydrogen atoms added. If the selected atom has two or
more bonds, it is replaced with the new atom type, but bond distances are not changed and additional
hydrogen atoms are not added.

Hydrogen is an exception to this pattern. If H is selected on the periodic table, clicking on any atom
will add a single H atom (it will not replace the atom with H).

### Adding Metals

Initially, only a subset of the periodic table is shown, with main group elements (excluding the noble gases)
displayed. To add any of the remaining elements, select the **Metals** link above Oxygen/Fluorine.
To revert back to showing only main group elements, select the **Organic** link.

### Deleting Atoms

To delete an atom, select the
 **[Delete Atom]**
button, then click on the atom to be removed. This atom and any hydrogen atoms attached to this atom
should be removed.

### Adding Bonds

To add a bond, place the pointer on the first atom and press down (but do not release). Drag the pointer
to the second atom, then release the mouse. A bond should be shown connecting these atoms. Note that when
adding bonds, the number of bonded atoms increases, so it may be necessary to delete one or more atoms.

### Removing Bonds

Deleting bonds is done in a similar manner. Select
 **[Delete Bond]**,
then place the pointer on the first atom and press down (but do not release). Drag the pointer
to the second atom, then release the mouse. The bond connecting these atoms should be removed.

### Rotating Around a Bond

It is also possible to rotate around bonds. To do this, first press the
 **[Rotate Bond]**
button. Then move the mouse pointer over the first atom, press and hold the mouse button, "drag" to
the second bonded atom and release the mouse button. (Note that if the atoms are not bonded, rotation
will not behave as expected). A new view of the molecule will be displayed with the molecule oriented
so that you are looking "down" the selected bond. The second atom selected will be in front, eclipsing
the first atom selected.

To rotate around this bond, use the mouse pointer to select a blank portion of the drawing window and
"drag" the pointer while holding the mouse button down. To get out of bond rotation mode, press the
 **[Rotate Bond]**,
which should change color to indicate that it is no longer active.

### Undo

When significant changes are made to the molecule (atoms added, atoms deleted, rotation about bonds, etc.),
the molecular coordinates are saved. Currently, ten sets of saved coordinates are saved. Pressing the
 **[Undo]**
button restores the most recent set of coordinates. This may be repeated up to the the limit of saved
sets of coordinates. There is also a
 **[Redo]**
button, that reverses the effects of the **Undo**.

### Structure Optimization

At the bottom of the Draw mode window is an
 **[Optimize Structure]**
button. This button will cause a crude geometry optimization to be performed. It is not necessary to use
this, but it can be useful when significant changes have occurred. This is most common when H atoms have
been added or when bonds have been added or removed. At this point, the optimization routine is very crude,
and it may be necessary to optimize a structure multiple times before a reasonable structure is obtained.

# File Format for Molecules

Currently, the only format supported by this program for reading molecular coordinates is the .xyz format.
These files can be created using the Open Babel program. Sample files
in this format are available in the molecules directory.

### Example

On the default page is a
 **[Show coordinates]**
button that displays coordinates for the current molecule in the information box at the bottom of the
screen. An example of the output produced for the methane molecule is:

```
5
CH4   (16.04 g/mol) in xyz format: From CH5M3D
C         0.0000       0.0000       0.0000
H         0.8740       0.6180       0.0000
H        -0.8740       0.6180       0.0000
H         0.0000      -0.6180       0.8740
H         0.0000      -0.6180      -0.8740
```

The first line contains the number of atoms in the molecule.

The second line is a comment. This line must be present, but can be blank or contain any text.

The remaining lines contain the elemental symbol (1-3 characters) and x, y, z cartesian coordinates. All of these
values are separated by one or more spaces.

This program attempts to be flexible in reading these files, and does not require data to be present in specific
columns. The one restriction that is enforced is that files must be named with the extension ".xyz".

# Description of Variations

The following files are provided with this distribution to illustrate different ways that this interface
can be used.

### Pre-load

This simple page loads and displays the structure of a molecule from a file stored on the server. The name of
this file is part of the web page html and cannot be changed by the user. While the molecule can be rotated
and information displayed, the user cannot change this structure.

### Chooser

This page allows the user to select the file to be viewed from a list of files stored on the server using
either buttons or from a drop-down select list. While the molecule can be rotated and information displayed,
the user cannot alter any of these structures.

### Gallery

This page loads a list of files from the server and displays each of these in a separate division along with a
description. Each of the molecules can be rotated independently and information displayed. However, the user
cannot change any of these structures.

### Viewer (only)

This page allows loading and viewing of molecules from files stored on the user's computer, but does not allow
for any editting of these structures.

### View 2 Windows

This page illustrates that more than one molecule can be loaded on a page. This page also does not allow
for any editting of either structure. To switch between active windows, use the mouse to click on any portion
of a drawing canvas.

### Two Windows

This page illustrates that more than one molecule can be loaded on a page, and that these windows do not have to
be the same size. In this view, both **View Mode** and **Draw Mode** are enabled, so
either (or both) of the structures being displayed can be altered.

### Javascript

This page illustrates how a user might create a simple function that interacts with functions contained within
the CH5M3D library to gather information about the active molecule and interact with (alter) this structure. In
this example, mirror images of a chiral molecule are generated with the user chosing the mirror plane to use.

### Quantum Chemistry Interface

This page illustrates a simple interface that has been used to create input files for the quantum mechanical program
GAMESS.
This page makes calls to several PHP files, and uses a very simple authorization scheme. To view this page, use the
username "**admin**" and the password "**password**".

Because this interface makes calls to the underlying operating system, it is unlikely that this interface will work
without modification. All of the files used to create this page are located in the qchem subdirectory.
