# Supplementary material for: CH5M3D: an HTML5 program for creating 3D molecular structures
Source: J Cheminform. 2013 Nov 18;5:46. doi: 10.1186/1758-2946-5-46 (PMC4177146; doi:10.1186/1758-2946-5-46)
Supplement: Additional file 1 — This archive contains all of the files required to create a fully-functional website using the CH5M3D library. [file 1758-2946-5-46-S1.zip › ch5m3d/doc/installation.html]

CH5M3D


CH5M3D

- CH5M3D Home
- Documentation
  - Introduction
  - Installation
  - Web Browsers
  - User Interface
  - Keyboard/Mouse
  - Drawing
  - File Format
  - PDF Manual
- Variations
  - Description
  - Pre-Load
  - Chooser
  - Gallery
  - Viewer (only)
  - View 2 Windows
  - Two Windows
  - Javascript
  - Quantum Interface
- Information
  - About
  - Project Homepage
  - Library API Info
  - GNU License

# Installation

This "program" can be downloaded as a single zip file containing the following files.

- **index.html** - The main HTML file loaded by a web browser.
- **ch5m3d.js** - The javascript code required to generate the web interface.
- **ch5m3d.css** - CSS code controlling the appearance of the web pages.
- **documentation.pdf** - A PDF version of the documentation.
- **doc/** - A directory containing html documentation files and the GNU license.
- **molecules/** - A directory containing a small number of .xyz formatted
  files of molecular coordinates.
- **variations/** - A directory containing a few html files that illustrate different ways
  that web pages can use the javascript code to provide different views.
- **qchem/** - A directory containing a php files and support files that provide a simple
  front-end interface to the quantum mechanical program GAMESS. (Note that it is very unlikely that this
  will work without modification).

### Testing

A live version of this program is available at the
Project Web page on SourceForge. Note that a
suitable web browser that supports HTML5 is required to run this program.

### Installation on a Web Server

No special steps are required to install this program on a web server other than placing the "unzipped"
directory tree in a location where it can be accessed by the web server. All file locations are coded
as "relative" directories, so the actual path/directory location for these files does not matter.

If it is desired to change the relative location of any of these files, note that **index.html**
assumes that **ch5m3d.js** and **ch5m3d.css** are both located in the same directory
as **index.html**. All of the documentation files assume that **ch5m3d.css**
is located "up" one directory (../ch5m3d.css).

The chem3d.js library copyright © 2013 by Clarke Earley  
and is distributed under the terms of the
GNU General Public License.
