# Supplementary material for: CH5M3D: an HTML5 program for creating 3D molecular structures
Source: J Cheminform. 2013 Nov 18;5:46. doi: 10.1186/1758-2946-5-46 (PMC4177146; doi:10.1186/1758-2946-5-46)
Supplement: Additional file 1 — This archive contains all of the files required to create a fully-functional website using the CH5M3D library. [file 1758-2946-5-46-S1.zip › ch5m3d/doc/about.html]

CH5M3D


CH5M3D

- CH5M3D Home
- Documentation
  - Introduction
  - Installation
  - Web Browsers
  - User Interface
  - Keyboard/Mouse
  - Drawing
  - File Format
  - PDF Manual
- Variations
  - Description
  - Pre-Load
  - Chooser
  - Gallery
  - Viewer (only)
  - View 2 Windows
  - Two Windows
  - Javascript
  - Quantum Interface
- Information
  - About
  - Project Homepage
  - Library API Info
  - GNU License

**CH5M3D Version 1.2.5**

Project Homepage

### Description

This program uses a combination of HTML5 and javascript to interactively draw 3-dimensional
structures of small molecules.

### License

This program is free software: you can redistribute it and/or modify it under the terms of the
GNU General Public License as published by the Free Software Foundation, either version 3 of the
License, or (at your option) any later version.

This program is distributed in the hope that it will be useful, but WITHOUT ANY WARRANTY; without even
the implied warranty of MERCHANTABILITY or FITNESS FOR A PARTICULAR PURPOSE. See the
GNU General Public License for more details.

To view a copy of the GNU General Public License, see
http://www.gnu.org/licenses/gpl.html.

### Author

Dr. Clarke Earley  
Associate Professor of Chemistry  
Kent State University at Stark  
cearley@kent.edu

### Acknowledgements

The support of Kent State University at Stark is gratefully acknowledged.

The chem3d.js library copyright © 2013 by Clarke Earley  
and is distributed under the terms of the
GNU General Public License.
