# Supplementary material for: CH5M3D: an HTML5 program for creating 3D molecular structures
Source: J Cheminform. 2013 Nov 18;5:46. doi: 10.1186/1758-2946-5-46 (PMC4177146; doi:10.1186/1758-2946-5-46)
Supplement: Additional file 1 — This archive contains all of the files required to create a fully-functional website using the CH5M3D library. [file 1758-2946-5-46-S1.zip › ch5m3d/doc/apidoc.html]

CH5M3D


CH5M3D

- CH5M3D Home
- Documentation
  - Introduction
  - Installation
  - Web Browsers
  - User Interface
  - Keyboard/Mouse
  - Drawing
  - File Format
  - PDF Manual
- Variations
  - Description
  - Pre-Load
  - Chooser
  - Gallery
  - Viewer (only)
  - View 2 Windows
  - Two Windows
  - Javascript
  - Quantum Interface
- Information
  - About
  - Project Homepage
  - Library API Info
  - GNU License

### function element(Z,param)

Define public routine to store and return elemental information

Parameters:

Z = Atomic number of element

param:

  symbol - Elemental symbol

  block - "s", "p", "d", or "f" block

  valence - # of valence electrons

  mass - Atomic mass

  radius - Covalent radius of atom

  EN   - Electronegativity of atom

  color - color of atom (based on JMol)

  gradient - gradient color for atom

  label - color for label

Return value:

Variable containing requested information

### function drawPeriodic()

Writes html code to a division named "ptable". The periodic table is written as a table, and each element

is linked to to the pickElem() routine.

### function Mol(value)

Define public routine to store and return molecule arrays

Parameter:

value = If value given, then set as active molecule

Return value:

Pointer to current array for molecular coordinates, ex.

var molecule = Mol();

Molecular values stored in 'zeroth' element of array

molecule[0].molIndex - ID number for this molecule

molecule[0].numatoms - # of atoms in this molecule

molecule[0].AtomScale - Scale factor to control size of molecule displayed

molecule[0].showlabels - 0=don't show elemental symbols, 1=show symbols

molecule[0].showcharges - 0=don't show charges, 1=show charges

molecule[0].gradients - 0=don't shade atoms, 1=use shading

molecule[0].formula - Simple text string containing molecular formula

molecule[0].weight   - Molecular weight

molecule[0].charge   - Charge of molecule

Atomic values in remaining elements of array

molecule[i].atomicnumber - Atomic number (Z) for atom

molecule[i].x     - x coordinate of atom

molecule[i].y     - y coordinate of atom

molecule[i].z     - z coordinate of atom

molecule[i].charge   - Estimate of atomic charge

molecule[i].highlite - 0=don't highlight atom, 1=highlight atom

molecule[i].hide   - 0=display atom, 1=do not display atom

### function BondMatrix(value)

Define public routine to store and return Bond Matrix

Parameter:

value = If value given, then set as active matrix

Return value:

Pointer to current bond matrix, ex.

var bonds = BondMatrix();

bonds[i][j] then gives indication of bond between atoms "i" and "j"

### function lookupSymbol(symbol)

Given elemental symbol, lookup and return atomic number (Z)

### function addAtom(AtomicNum, x, y, z)

Routine to add atomic information to molecule array.

Parameters are the atomic # and (x,y,z) coordinates of the atom.

### function addBond(atom1, atom2)

Routine to add bonding information to bonds array

(Note that atom numbers start at 1)

### function delAtom(atomNum)

Routine to remove selected atom, directly-bonded Hydrogens, and

cleanup molecule object (remove blank slots) and update bond arrays.

atomNum is the position of the atom to delete in the molecule array

### function delBond(atom1, atom2)

Routine to remove bond from bonds array.

### function hideH()

Do not display any hydrogen atoms in molecule

### function showAll()

Show all atoms in molecule

### function centerMolecule()

Find center of molecule and move coordinates to center.

### function showCoord(mode)

Display molecular coordinates and bonds in information window.

Parameter:

mode=0: Write .xyz formatted file

mode>0: Write coordinates and bond information

### function formula()

Display molecular formula to division with an id = "formula".

Note that the contents of this division will be overwritten.

### function RotateMolecule(axis)

Routine to start/stop rotation of molecule.

Parameter:

axis = rotation axis. Allowed values are "x", "y", or "z"

  ("s" can be used to stop all rotations)

Buttons must be created in html with:

id="rotateX" (or rotateY or rotateZ)

### function drawMolecule()

Draw atoms and bonds.

### function showLabels()

Routine to toggle display of atomic labels.

### function parameters()

Define constants used to control drawing.

Get/set values using:

var param = parameters();

param.mode = Interface mode. Set to "Draw" or "View"

param.element = Type of element to add next. Default = "C"

param.clouds = # of hybrid orbitals. Default = 4

param.bondmode = Bond mode. Set to "Add", "Delete", or "Rotate"

param.atommode = Add atom mode. Set to "Add" or "Delete"

### function drawmode()

Routine to enable display of "draw mode" interface.

### function viewmode()

Routine to enable display of "view mode" interface.

### function setCharge()

Routine to set the molecular charge using value of select form with id="SelectCharge".

### function simpleQ()

Public routine to (somewhat arbitrarily) assign electron configuration,

including bond orders, and calculate the charges on each atom.

### function showBondMatrix(BondMtx,BondWin)

Write Bond information to output window.

### function showgallery(title,base,List,Desc,size)

Routine to display multiple files stored on the web server.

Parameters:

title: Title to display before gallery

  (If title = "delete", clear gallery)

base: Path to directory (relative to web root) containing images

List: Array containing a list of filenames (including extensions)

Desc: Array containing descriptions for each file

size: integer width (in px) for each frame

### function galleryUniform()

Routine to force all molecules that are part of a "gallery" to be displayed with the same size scale.

### function galleryReset()

For molecules that are part of a "gallery", this routine optimizes the size of each molecule individually.

### function readServerFile(filename)

Routine to read contents of file stored on the web server.

Parameter:

filename: Name (URL) of file (including path)

### function InfoWin(mytext,mode)

Routine to write text to information window (textarea with an id of "information").

Parameters:

mytext: String to write to output window

mode: If >0, then clear text window

### function loadMolecule()

Routine to load molecular information for methane molecule.

### function resetView()

Routine to reset view. Center, rescale, and remove highlights.

### function buttonColor(button, mode)

Set color of buttons

Parameters

button - string containing ID name for button

mode - 0=inactive, 1=active

### function initialize()

Initialization Routines

- Load properties of elements

- Define Handlers for mouse events

- Draw molecule

### function mechanics()

Simple routine to perform crude optimization of structure.

### function distance(mol, atomA, atomB)

Calculate the distance between two atoms in mol array.

### function angle(mol, atomA, atomB, atomC)

Calculate the angle between A--B--C in mol array

### function dihedral(mol, atomA, atomB, atomC, atomD)

Routine to calculate the dihedral angle for A--B--C--D

The chem3d.js library copyright © 2013 by Clarke Earley  
and is distributed under the terms of the
GNU General Public License.
