# Supplementary material for: CH5M3D: an HTML5 program for creating 3D molecular structures
Source: J Cheminform. 2013 Nov 18;5:46. doi: 10.1186/1758-2946-5-46 (PMC4177146; doi:10.1186/1758-2946-5-46)
Supplement: Additional file 1 — This archive contains all of the files required to create a fully-functional website using the CH5M3D library. [file 1758-2946-5-46-S1.zip › ch5m3d/doc/view.html]

CH5M3D


CH5M3D

- CH5M3D Home
- Documentation
  - Introduction
  - Installation
  - Web Browsers
  - User Interface
  - Keyboard/Mouse
  - Drawing
  - File Format
  - PDF Manual
- Variations
  - Description
  - Pre-Load
  - Chooser
  - Gallery
  - Viewer (only)
  - View 2 Windows
  - Two Windows
  - Javascript
  - Quantum Interface
- Information
  - About
  - Project Homepage
  - Library API Info
  - GNU License

# User Interface: View Mode

The image below shows the initial view that should be presented when this page is first loaded.
The most important sections are labeled in red. Upon initially loading this page, you should be
in **View Mode**, indicated by both the highlighted
tab and the text "View Mode" displayed below this button. You can switch between
**View Mode** and **Draw Mode** at any time by selecting the appropriate tab.

Initially, the methane molecule (CH4) should be displayed in the
**Drawing Window**. While in View mode, you can use your mouse/pointer to perform the
following tasks.

- **Rotate Molecule** - Move the pointer to any blank portion of the Drawing Window
  and hold the mouse button down. Dragging the pointer should cause the molecule to rotate. To stop
  rotation, simply release the mouse button. Simultaneously pressing **<Shift>**
  while dragging the mouse pointer will cause the molecule to rotate around the z-axis. It is
  also possible to 'translate' a molecule by pressing the **<Ctrl>** key
  while dragging the mouse.
- **Zoom** - If a mouse scroll wheel is available, this can be used to either zoom-in
  or zoom-out the current view.
- **Identify Atom** - Move the pointer over any atom in a molecule and click on this
  atom to select. The Elemental symbol followed by a number should be displayed. The number is the
  position of the atom in the set of coordinates for this molecule, starting at 1. If the
  button is active, then the calculated charge on this atom will also be displayed.
- **Highlight Atom** - Move the pointer over any atom in a molecule, hold down the
  **<Shift>** key and click on this atom. A semitransparent yellow circle should
  appear over this atom. <Shift>-click on this atom a second time to turn off the highlight.
  Any number of atoms may be highlighted. The
  button may also be used to remove all highlights.
- **Measure Bond Lengths** - Move the pointer to the first atom of the bond and select.
  The label for this atom should appear in the upper-right corner of the display window. Then, move
  the pointer to the second atom and select this atom. The bond length (in Angstroms) will be shown
  at the top right of the drawing window.
- **Measure Bond Angles** - By selecting three atoms, the bond angle (in degrees)
  around the central atom (the second atom selected) will be shown.
- **Measure Dihedral Angles** - By selecting four atoms, the dihedral angle
  (in degrees) will be shown. For example, if all four atoms lie in a plane, the dihedral angle will
  be either 0° or 180°.

For all of the operations, clicking on a blank portion of the screen will clear the list of selected
atoms and allow you to start measuring a different set of lengths/angles. Clicking on the same atom
twice will cause the atom list to be reset and this selected atom will be placed as the first atom on
the list.

To the right of the **Drawing Window** are several buttons. These provide the following
options:

- This button will print the coordinates in .xyz format in the **Information Window**.
  For security reasons, it is not possible for javascript to write files to a user's computer.
  To save the coordinates of molecules created using this program, it is required that users open a
  text editor, copy the coordinates from the **Information Window** into their editor
  and save this as an .xyz file. When saving this file, but sure that it is saved as an unformatted
  text file.
- This button toggles the display of labels (elemental symbols) for each atom.
- Selecting this button causes a crude computation of atomic charge to be performed. Charges are shown
  as semi-transparent spheres around each atom. Negative charges are shown in red, and positive charges
  are shown as blue spheres. The intensity of these colors is used to indicate the magnitude of the charges.
  This charge calculation is based on a combination of formal charges and electronegativity differences.
  In addition, the dipole moment of the molecule based on these approximate point charges is displayed in
  the Information window near the bottom of the screen. Note that these calculated charges should *not*
  be considered to be accurate values but instead treated as a crude, first approximation.
- This button centers and rescales the molecule to fit the display, and removes all highlights.
- By selecting this button, users can load .xyz formatted files. Files of this format can be generated
  using the Open Babel program. A few sample .xyz files
  are available in the **molecules** folder on the
  CH5M3D Web site.

To **save an image** of a molecule, first rotate the molecule to get it into the desired
orientation. Then, perform a right-click using the mouse pointer and select "Save Image As...".

The chem3d.js library copyright © 2013 by Clarke Earley  
and is distributed under the terms of the
GNU General Public License.
