# Supplementary material for: CH5M3D: an HTML5 program for creating 3D molecular structures
Source: J Cheminform. 2013 Nov 18;5:46. doi: 10.1186/1758-2946-5-46 (PMC4177146; doi:10.1186/1758-2946-5-46)
Supplement: Additional file 1 — This archive contains all of the files required to create a fully-functional website using the CH5M3D library. [file 1758-2946-5-46-S1.zip › ch5m3d/doc/drawing.html]

CH5M3D


CH5M3D

- CH5M3D Home
- Documentation
  - Introduction
  - Installation
  - Web Browsers
  - User Interface
  - Keyboard/Mouse
  - Drawing
  - File Format
  - PDF Manual
- Variations
  - Description
  - Pre-Load
  - Chooser
  - Gallery
  - Viewer (only)
  - View 2 Windows
  - Two Windows
  - Javascript
  - Quantum Interface
- Information
  - About
  - Project Homepage
  - Library API Info
  - GNU License

# Drawing Molecules

In Draw Mode, you have the option of adding or deleting atoms and/or bonds to any structure shown in the
display window. The image below shows the buttons initially displayed when first entering Draw Mode.

### Rotating the Entire Molecule

In draw mode, you can rotate the molecule in the same manner as performed in View mode by selecting a
blank portion of the screen and "dragging" the pointer.

### Adding Atoms

Initially, a subset of the periodic table is shown, with Carbon highlighted. To add a methyl group to the
methane molecule shown, click on any of the hydrogen atoms. By default, the atom added is assumed to be
sp3 hybridized. To add a CH2 group, select
before clicking on an H.

In general, clicking on any atom will convert the atom into the selected element type shown on the
periodic table. If the selected atom has only one bond, the new atom is added with a reasonable value
for the bond length and the appropriate number of hydrogen atoms added. If the selected atom has two or
more bonds, it is replaced with the new atom type, but bond distances are not changed and additional
hydrogen atoms are not added.

Hydrogen is an exception to this pattern. If H is selected on the periodic table, clicking on any atom
will add a single H atom (it will not replace the atom with H).

### Adding Metals

Initially, only a subset of the periodic table is shown, with main group elements (excluding the noble gases)
displayed. To add any of the remaining elements, select the **Metals** link above Oxygen/Fluorine.
To revert back to showing only main group elements, select the **Organic** link.

### Deleting Atoms

To delete an atom, select the
button, then click on the atom to be removed. This atom and any hydrogen atoms attached to this atom
should be removed.

### Adding Bonds

To add a bond, place the pointer on the first atom and press down (but do not release). Drag the pointer
to the second atom, then release the mouse. A bond should be shown connecting these atoms. Note that when
adding bonds, the number of bonded atoms increases, so it may be necessary to delete one or more atoms.

### Removing Bonds

Deleting bonds is done in a similar manner. Select
,
then place the pointer on the first atom and press down (but do not release). Drag the pointer
to the second atom, then release the mouse. The bond connecting these atoms should be removed.

### Rotating Around a Bond

It is also possible to rotate around bonds. To do this, first press the
button. Then move the mouse pointer over the first atom, press and hold the mouse button, "drag" to
the second bonded atom and release the mouse button. (Note that if the atoms are not bonded, rotation
will not behave as expected). A new view of the molecule will be displayed with the molecule oriented
so that you are looking "down" the selected bond. The second atom selected will be in front, eclipsing
the first atom selected.

To rotate around this bond, use the mouse pointer to select a blank portion of the drawing window and
"drag" the pointer while holding the mouse button down. To get out of bond rotation mode, press the
,
which should change color to indicate that it is no longer active.

### Undo

When significant changes are made to the molecule (atoms added, atoms deleted, rotation about bonds, etc.),
the molecular coordinates are saved. Currently, ten sets of saved coordinates are saved. Pressing the
button restores the most recent set of coordinates. This may be repeated up to the the limit of saved
sets of coordinates. There is also a
button, that reverses the effects of the **Undo**.

### Structure Optimization

At the bottom of the Draw mode window is an
button. This button will cause a crude geometry optimization to be performed. It is not necessary to use
this, but it can be useful when significant changes have occurred. This is most common when H atoms have
been added or when bonds have been added or removed. At this point, the optimization routine is very crude,
and it may be necessary to optimize a structure multiple times before a reasonable structure is obtained.

The chem3d.js library copyright © 2013 by Clarke Earley  
and is distributed under the terms of the
GNU General Public License.
