# Supplementary material for: CH5M3D: an HTML5 program for creating 3D molecular structures
Source: J Cheminform. 2013 Nov 18;5:46. doi: 10.1186/1758-2946-5-46 (PMC4177146; doi:10.1186/1758-2946-5-46)
Supplement: Additional file 1 — This archive contains all of the files required to create a fully-functional website using the CH5M3D library. [file 1758-2946-5-46-S1.zip › ch5m3d/doc/introduction.html]

CH5M3D


CH5M3D

- CH5M3D Home
- Documentation
  - Introduction
  - Installation
  - Web Browsers
  - User Interface
  - Keyboard/Mouse
  - Drawing
  - File Format
  - PDF Manual
- Variations
  - Description
  - Pre-Load
  - Chooser
  - Gallery
  - Viewer (only)
  - View 2 Windows
  - Two Windows
  - Javascript
  - Quantum Interface
- Information
  - About
  - Project Homepage
  - Library API Info
  - GNU License

# CH5M3D Overview

This web interface has been developed to allow users to create and visualize 3-dimensional drawings of simple
molecules without requiring the download of any additional software. In addition to creating structures, users
can also load existing XYZ formatted files (such as those generated by
Open Babel) containing 3-dimensional coordinates. A small
selection of .xyz formatted files is available from the
project home page.

Once molecules are created or read from a file, users can examine geometry information (bond lengths, angles,
and dihedral angles) and modify these structures. Routines to perform a crude geometry optimization and a simple
calculation of atomic charges are also available.

This web interface relies on HTML5 (in particular, the HTML5 Canvas), and as such requires a
modern web browser to run. The advantage of this approach is the no additional
plugins or Java is required to use this program. Support for mobile devices is currently only partially implemented,
but is planned to be included in future versions.

### Screenshots

Initial view of CH5M3D Interface

Image with labels added

Image with highlighting

Draw Mode interface

Example of charge calculation

The chem3d.js library copyright © 2013 by Clarke Earley  
and is distributed under the terms of the
GNU General Public License.
