# Supplementary material for: CH5M3D: an HTML5 program for creating 3D molecular structures
Source: J Cheminform. 2013 Nov 18;5:46. doi: 10.1186/1758-2946-5-46 (PMC4177146; doi:10.1186/1758-2946-5-46)
Supplement: Additional file 1 — This archive contains all of the files required to create a fully-functional website using the CH5M3D library. [file 1758-2946-5-46-S1.zip › ch5m3d/doc/browser.html]

CH5M3D


CH5M3D

- CH5M3D Home
- Documentation
  - Introduction
  - Installation
  - Web Browsers
  - User Interface
  - Keyboard/Mouse
  - Drawing
  - File Format
  - PDF Manual
- Variations
  - Description
  - Pre-Load
  - Chooser
  - Gallery
  - Viewer (only)
  - View 2 Windows
  - Two Windows
  - Javascript
  - Quantum Interface
- Information
  - About
  - Project Homepage
  - Library API Info
  - GNU License

# Supported Web Browsers

**Your browser does NOT appear to support the required HTML5 Canvas element.**

**Your browser appears to support the required HTML5 features.**

The interactive drawing window used on this website requires HTML5, which is NOT supported by version 8
or lower of Microsoft Internet Explorer. To use this site, you will need to use a browser that
supports the Canvas element and a few other features of HTML5. Listed below are several freely available
web browsers that should work with this interface.

- Mozilla Firefox - Available for Microsoft Windows,
  Apple, Linux, and Android.
- Google Chrome - Available for Microsoft Windows,
  Apple, Linux, Android, and iOS.
- Opera - While not as popular, is available for Microsoft
  Windows, Apple, Linux, Android, and iOS.
- Apple Safari - Should already be installed on current Mac
  computers.

Currently, touch screen devices are NOT fully supported by this interface. While the web page will load, several
important tasks do not function properly on tablets or other touch screen devices.

The chem3d.js library copyright © 2013 by Clarke Earley  
and is distributed under the terms of the
GNU General Public License.
