# Supplementary material for: CH5M3D: an HTML5 program for creating 3D molecular structures
Source: J Cheminform. 2013 Nov 18;5:46. doi: 10.1186/1758-2946-5-46 (PMC4177146; doi:10.1186/1758-2946-5-46)
Supplement: Additional file 1 — This archive contains all of the files required to create a fully-functional website using the CH5M3D library. [file 1758-2946-5-46-S1.zip › ch5m3d/doc/description.html]

CH5M3D


CH5M3D

- CH5M3D Home
- Documentation
  - Introduction
  - Installation
  - Web Browsers
  - User Interface
  - Keyboard/Mouse
  - Drawing
  - File Format
  - PDF Manual
- Variations
  - Description
  - Pre-Load
  - Chooser
  - Gallery
  - Viewer (only)
  - View 2 Windows
  - Two Windows
  - Javascript
  - Quantum Interface
- Information
  - About
  - Project Homepage
  - Library API Info
  - GNU License

# Description of Variations

The following files are provided with this distribution to illustrate different ways that this interface
can be used.

### Pre-load

This simple page loads and displays the structure of a molecule from a file stored on the server. The name of
this file is part of the web page html and cannot be changed by the user. While the molecule can be rotated
and information displayed, the user cannot change this structure.

### Chooser

This page allows the user to select the file to be viewed from a list of files stored on the server using
either buttons or from a drop-down select list. While the molecule can be rotated and information displayed,
the user cannot alter any of these structures.

### Gallery

This page loads a list of files from the server and displays each of these in a separate division along with a
description. Each of the molecules can be rotated independently and information displayed. However, the user
cannot change any of these structures.

### Viewer (only)

This page allows loading and viewing of molecules from files stored on the user's computer, but does not allow
for any editting of these structures.

### View 2 Windows

This page illustrates that more than one molecule can be loaded on a page. This page also does not allow
for any editting of either structure. To switch between active windows, use the mouse to click on any portion
of a drawing canvas.

### Two Windows

This page illustrates that more than one molecule can be loaded on a page, and that these windows do not have to
be the same size. In this view, both **View Mode** and **Draw Mode** are enabled, so
either (or both) of the structures being displayed can be altered.

### Javascript

This page illustrates how a user might create a simple function that interacts with functions contained within
the CH5M3D library to gather information about the active molecule and interact with (alter) this structure. In
this example, mirror images of a chiral molecule are generated with the user chosing the mirror plane to use.

### Quantum Chemistry Interface

This page illustrates a simple interface that has been used to create input files for the quantum mechanical program
GAMESS.
This page makes calls to several PHP files, and uses a very simple authorization scheme. To view this page, use the
username "**admin**" and the password "**password**".

Because this interface makes calls to the underlying operating system, it is unlikely that this interface will work
without modification. All of the files used to create this page are located in the qchem subdirectory.

The chem3d.js library copyright © 2013 by Clarke Earley  
and is distributed under the terms of the
GNU General Public License.
