# Supplementary material for: CH5M3D: an HTML5 program for creating 3D molecular structures
Source: J Cheminform. 2013 Nov 18;5:46. doi: 10.1186/1758-2946-5-46 (PMC4177146; doi:10.1186/1758-2946-5-46)
Supplement: Additional file 1 — This archive contains all of the files required to create a fully-functional website using the CH5M3D library. [file 1758-2946-5-46-S1.zip › ch5m3d/index.html]

CH5M3D


CH5M3D

- Documentation
  - Introduction
  - Installation
  - Web Browsers
  - User Interface
  - Keyboard/Mouse
  - Drawing
  - File Format
  - PDF Manual
- Variations
  - Description
  - Pre-Load
  - Chooser
  - Gallery
  - Viewer (only)
  - View 2 Windows
  - Two Windows
  - Javascript
  - Quantum Interface
- Information
  - About
  - Project Homepage
  - Library API Info
  - GNU License

**View Mode**

Formula =

+3
+2
+1
0
-1
-2
-3

**Load file:**

**Current Element:**

**Hybridization:**

**Information**

The chem3d.js library copyright © 2013 by Clarke Earley  
and is distributed under the terms of the
GNU General Public License.
